# Biochemical Characterization and Cold-Adaption Mechanism of a PL-17 Family Alginate Lyase Aly23 from Marine Bacterium *Pseudoalteromonas* sp. ASY5 and Its Application for Oligosaccharides Production

**DOI:** 10.3390/md20020126

**Published:** 2022-02-06

**Authors:** Xiang Tang, Chao Jiao, Yi Wei, Xiao-Yan Zhuang, Qiong Xiao, Jun Chen, Fu-Quan Chen, Qiu-Ming Yang, Hui-Fen Weng, Bai-Shan Fang, Yong-Hui Zhang, An-Feng Xiao

**Affiliations:** 1 College of Food and Biological Engineering, Jimei University, Xiamen 361021, China; xiangyi@jmu.edu.cn (X.T.); jiaochao1@foxmail.com (C.J.); yiwei@jmu.edu.cn (Y.W.); zxy@jmu.edu.com (X.-Y.Z.); xiaoqiong129@jmu.edu.cn (Q.X.); chenjun@jmu.edu.cn (J.C.); fqchenhy0109@jmu.edu.cn (F.-Q.C.); yangqm@jmu.edu.cn (Q.-M.Y.); wenghuifen@jmu.edu.cn (H.-F.W.); 2 Fujian Provincial Engineering Technology Research Center of Marine Functional Food, Xiamen 361021, China; 3 Xiamen Key Laboratory of Marine Functional Food, Xiamen 361021, China; 4 College of Chemistry and Chemical Engineering, Xiamen University, Xiamen 361021, China; fbs@xmu.edu.cn

**Keywords:** alginate lyase, PL-17 family, cold-adapted, alginate oligosaccharides

## Abstract

As an important enzyme involved in the marine carbon cycle, alginate lyase has received extensive attention because of its excellent degradation ability on brown algae, which is widely utilized for alginate oligosaccharide preparation or bioethanol production. In comparison with endo-type alginate lyases (PL-5, PL-7, and PL-18 families), limited studies have focused on PL-17 family alginate lyases, especially for those with special characteristics. In this study, a novel PL-17 family alginate lyase, Aly23, was identified and cloned from the marine bacterium *Pseudoalteromonas carrageenovora* ASY5. Aly23 exhibited maximum activity at 35 °C and retained 48.93% of its highest activity at 4 °C, representing an excellent cold-adaptation property. Comparative molecular dynamics analysis was implemented to explore the structural basis for the cold-adaptation property of Aly23. Aly23 had a high substrate preference for poly β-D-mannuronate and exhibited both endolytic and exolytic activities; its hydrolysis reaction mainly produced monosaccharides, disaccharides, and trisaccharides. Furthermore, the enzymatic hydrolyzed oligosaccharides displayed good antioxidant activities to reduce ferric and scavenge radicals, such as hydroxyl, ABTS^+^, and DPPH. Our work demonstrated that Aly23 is a promising cold-adapted biocatalyst for the preparation of natural antioxidants from brown algae.

## 1. Introduction

Alginate, the most abundant carbohydrate in brown algae, is an acidic linear polysaccharide mainly obtained from the cell wall and intracellular material of brown seaweed [1], representing approximately 40% [2] of the dry weight of the plant. It is composed of (1,4)-linked β-D-mannuronic acid (M) and α-l-guluronic acid (G), and arranged as a poly β-d-mannuronate (polyM), poly α-L-guluronate (polyG), or heteropolymer [3]. Commercial alginates are mainly derived from algae, such as *Macrocystis*, *Laminaria*, and *Ascophyllum* [4]. Alginate is extensively used in food industries, biotechnology, and in medical applications [5] because it is highly viscous and gelatinous. In addition, alginate has been considered as a renewable alternative to fossil transport fuel to solve the energy problem [6]. It can reduce land consumption, as it can grow in seawater or industrial wastewater [7]. Alginate oligosaccharides (AOSs), the degradation products of alginate, have attracted considerable attention because of their remarkable biological activities in recent years. AOSs are useful for preserving fruit quality and enhancing shelf life by delayed abscisic acid accretion [8]. The anti-biofilm property of an AOS offers the potential for its use in biofilm-related bacterial diseases, such as cystic fibrosis and periodontal disease [9]. It can also be a superb natural antioxidant, which can be used to inhibit lipid oxidation in emulsion treatment, superior to ascorbic acid [10]. Apart from above activities, AOSs also have various physiological properties such as anti-tumor [11], immunomodulation [12], and anti-diabetic [13].

Alginate lyase, an important enzyme for the preparation of AOSs, catalyzes the degradation of alginate via β-elimination of the 4-O-glycosyl bond to form a double bond between C-4 and C-5. The 4-deoxy-L-erythro-hex-4-enopyranosyluronic residues are produced at the non-reducing end of the resulting oligosaccharides [14]. Alginate lyase has been detected from various sources, including marine mollusks, bacteria, fungi, and marine brown algae, some of which have been isolated and characterized [15]. Efficient alginate lyase is a key tool for the production of different functional AOSs from alginate [16]. At the same time, by saccharifying brown algae, the fermentable sugars’ components can be efficiently utilized and converted into bioethanol by biological platforms [17]. Therefore, it is of great significance to conduct a search for recombinant alginate lyases with specific properties that could be produced in large quantities [18]. In terms of the difference in their catalytic reaction mechanisms, alginate lyases are usually classified into endo- and exo-type enzymes. Generally, the degraded products of the exo-type lyase are monosaccharides that cleave the substrate from the chain ends [19]; the endo-type alginate lyase products are unsaturated alginate disaccharides, trisaccharides, and other oligosaccharides [20]. In addition, individual alginate lyases exhibit both endo- and exo-type activity [21]. Alginate lyases can be divided into several families of polysaccharide lyases (PL) as follows: PL-5, PL-6, PL-7, PL-14, PL-15, PL-17, PL-18, PL-31, PL-32, PL-36, and PL-39 [22]. However, compared with endo-type alginate lyases (PL-5, PL-7, and PL-18), the study of exo-type PL-17 family alginate lyases are still rather less, especially for those with special characteristics such as cold adaptability. Enzymes with cold adaptability can retain high catalytic activity at low temperatures, thus saving energy and expanding their application under low-temperature conditions. They can be easily inactivated by moderate temperatures to diminish their biological pollution. Nowadays, only a few alginate lyases present notable cold adaptability. Dong et al. [23] explored novel alginate lyase from the arctic ocean, in which 11 alginate lyases showed the highest activity at 20–30 °C among the strains isolated from the brown *alga Laminaria*. Gao et al. [5], reported that TsAly6A, obtained from *Thalassomonas* sp. LD5, exhibited cold adaptation, retaining 20% of the highest activity at 10 °C. Alginate lyase, AlyPM [24], from *Pseudoalteromonas* sp. SM0524, maintained 19% of the highest activity at 5 °C, and no detectable activity was observed at 45 °C.

In the present study, a novel PL-17 family alginate lyase, Aly23, was identified and cloned from marine bacterium *Pseudoalteromonas carrageenovora* ASY5, and expressed in *Escherichia coli*. Enzymatic characterization indicated that Aly23 is a new, cold-adapted PL-17 family alginate lyase. A preliminary investigation into the underlying structural basis for the cold-adaptation property of Aly23 was performed using comparative molecular dynamics (MD) analysis. Moreover, Aly23 hydrolyzed alginate to produce monosaccharides, disaccharides, and trisaccharides. The antioxidant capacity of the obtained AOS was also investigated. These results suggest that Aly23 would be a promising cold-adapted biocatalyst for industrial applications.

## 2. Results and Discussion

### 2.1. Cloning and Sequence Analysis of Aly23

**Figure 1:**
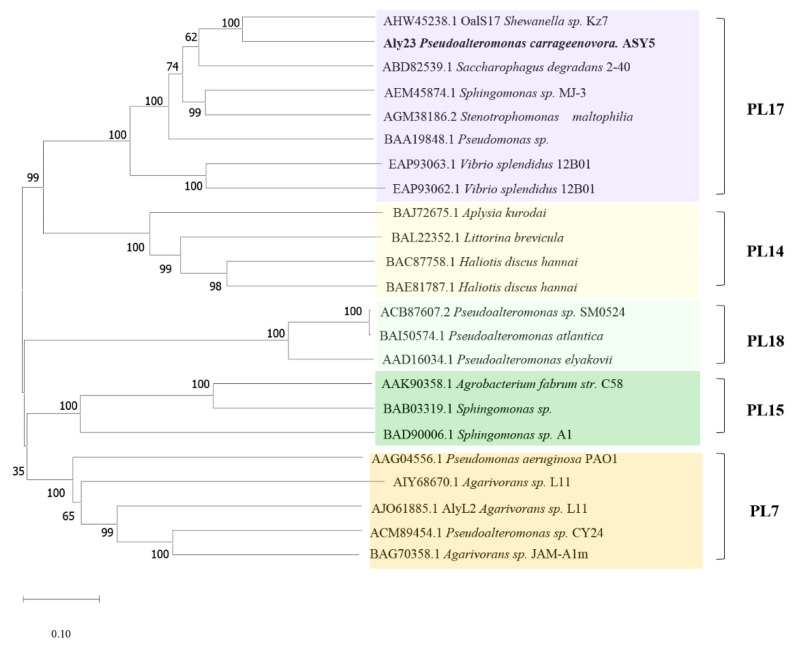
Phylogenetic analysis of Aly23 and related alginate lyases. The phylogenetic tree was generated with the neighbor-joining method by using Mega 7.0 software.

**Figure 2:**
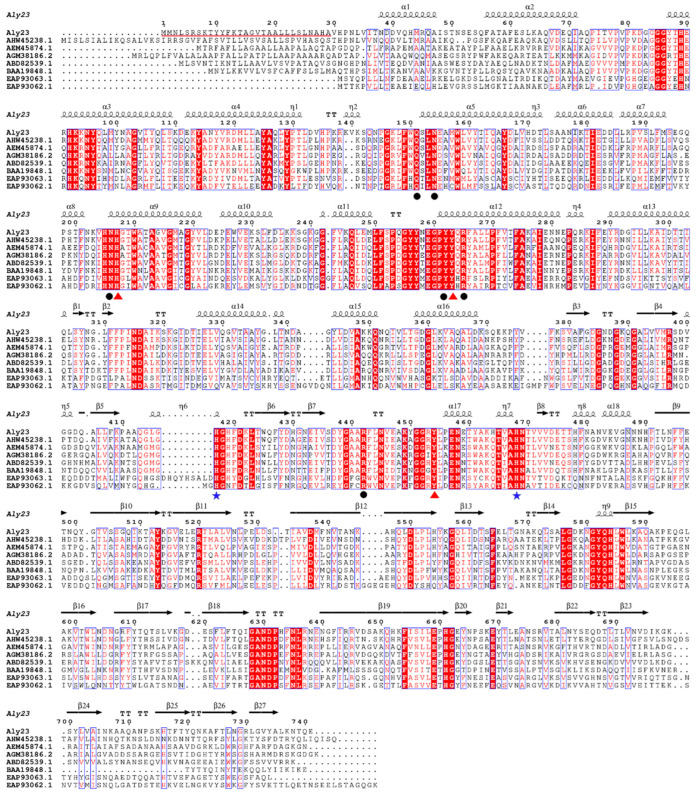
Sequence comparison of Aly23 with related alginate lyases from PL family 17. Assumed signal peptide marked with a red line. Conserved regions are highlighted with a red border. Residues involved in catalytic, metal-binding and substrate-binding pockets are shown by red triangles, a blue five-pointed star and a black dot, respectively.

The alginate lyase gene consisting of 2223 bp was cloned and sequenced (GenBank ID: QWT68652.1). Its open reading frame encoded a protein of 741 amino acids with a putative signal peptide (Met1-Ala29). The mature enzyme had a calculated molecular mass of 82.59 kDa and a predicted pI value of 6.55. To classify Aly23 alginate lyase, we constructed a phylogenetic tree (Figure 1) by using 23 characterized alginate lyases from 7, 14, 15, 17, and 18 PL families in the carbohydrate-active enzymes (CAZy) database. Therefore, Aly23 was an alginate lyase from *Pseudoalteromonas* sp. ASY5, belonging to the PL family 17. Aly23 was comprised of the AlgLyase superfamily domain (residue numbers 74–304) and Heparinase II/III-like protein domain (384–583). Sequence analysis of the PL-17 family of Aly23 and previously characterized alginate lyase are shown in Figure 2. The alignment shows that Aly23 was most homologous to alginate lyase from *Shewanella* sp. Kz7 (AHW45238.1) with an amino acid sequence identity of 55.87%. The catalytic amino acids (His207, Tyr263, and Tyr454), the metal-binding residues (His417 and His468) [25], and the carbohydrate binding amino acids (Gln151, Asn154, Asn206, Try262, Arg265, and Arg442) of PL-17 were completely conserved in the Aly23 sequence [26].

### 2.2. Expression and Purification of Recombinant Aly23

**Figure 3:**
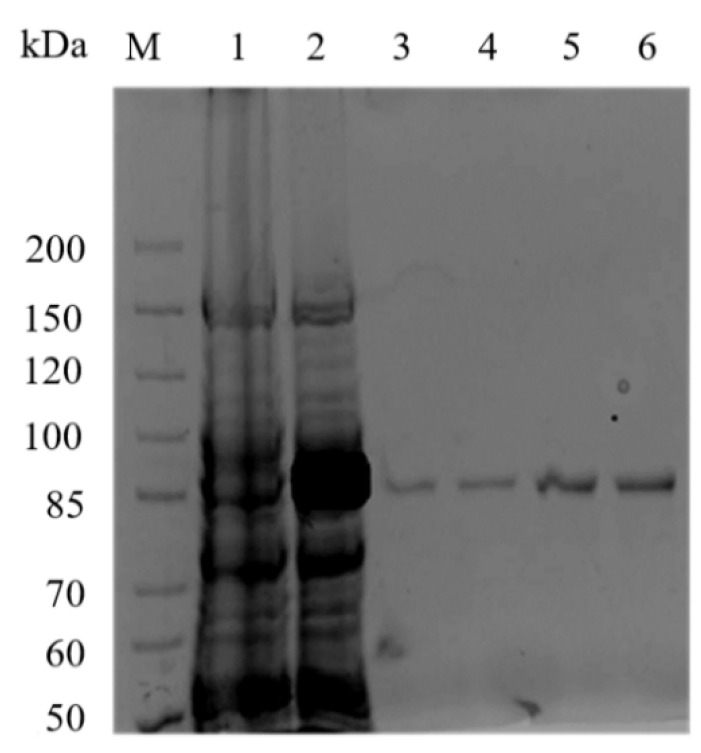
The SDS-PAGE profile of Aly23: M—protein marker; 1—total proteins from *E. coli* BL21(DE3) containing pET-28a-aly23 before induction; 2—total proteins from *E. coli* BL21(DE3) containing pET-28a (+) vector after induction with IPTG; 3–6—purified Aly23 from different tubes with different concentrations.

The recombinant strain was induced by adding 0.05 mmol/L isopropyl-beta-d-thiogalactopyranoside (IPTG) to express Aly23. Sodium dodecyl sulfate polyacrylamide gelelectrophoresis (SDS-PAGE) analysis (Figure 3) indicated that the crude extraction from *E. coli* BL21 (DE3), induced by IPTG, had a high-level expression of recombinant protein band compared with that without IPTG induction. The crude extract was purified in the Ni-NTA agarose column, and the activity of the purified Aly23 reached 6.76 U/mg. An SDS-PAGE analysis showed a single band at approximately 85 kDa, identical to the predicted molecular mass of the recombinant Aly23.

### 2.3. Biochemical Characterization of Aly23

**Figure 4:**
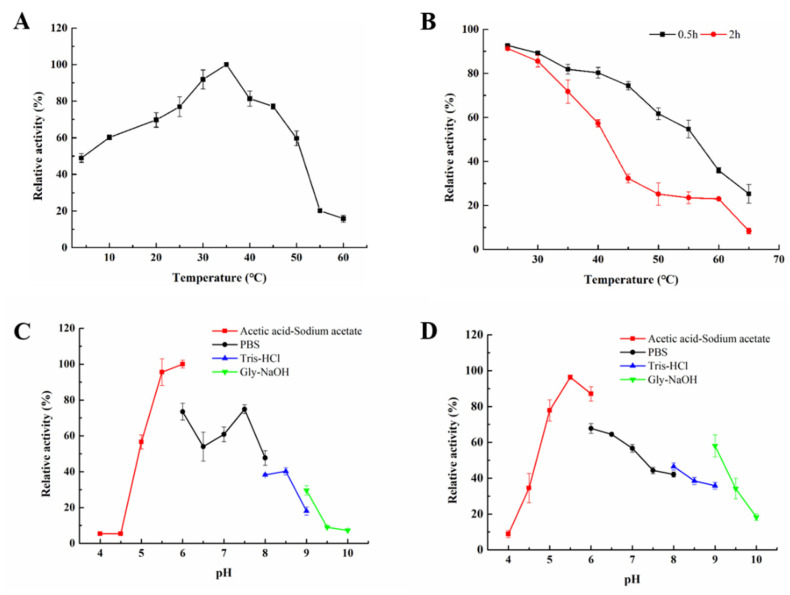
Effects of pH and temperature on the activity and stability of Aly23: (**A**) the optimal temperatures of the enzymes were determined based on the activity at various temperatures (4–60 °C); (**B**) thermostability of Aly23. The residual activity was determined at the optimal temperatures after incubation at various temperatures (25–65 °C) for 30 min or 2 h; (**C**) the optimal pH values of Aly23 were determined by measuring the activity in the acetic acid–sodium acetate (pH 4.0–6.0), Na_2_HPO_4_-NaH_2_PO_4_ (pH 6.0–8.0), Tris-HCl (pH 8.0–9.0) and glycine–sodium hydroxide buffer (pH 9.0–10.0); (**D**) pH stabilities of Aly23. The residual activity was measured after the enzyme was incubated in the pH at 4.0–10 with the above buffers for 24 h at 4 °C.

The comparison of the characteristics of PL-17 family alginate lyase is listed in Table 1. The optimal temperature and pH range for the reported PL-17 family alginate lyases were quite narrow, indicating that environmental factors significantly affected the activity. The optimal temperatures were between 30 and 50 °C, and the optimal pH was in the range of 6.0–8.0. The effects of temperature and pH on Aly23 were determined using sodium alginate as the substrate. The optimum temperature of the recombinant alginate lyase was determined over a temperature range of 4–60 °C. As shown in Figure 4A, Aly23 had the highest catalytic activity at 35 °C. Moreover, the thermostability was investigated at 25–65 °C. Aly23 was remarkably stable at 30 °C and was able to withstand incubation at 30 °C for 2 h, retaining 90% of its activity. Incubation at 45 °C for 30 min resulted in a 68% relative enzyme activity loss (Figure 4B). The results indicated that Aly23 is a cold-adapted alginate lyase with low optimal temperature and thermostability. Notably, compared with other cold-adaptation-characterized alginate lyases, Aly23 possessed high activity at a low temperature. The catalytic activity maintained 69.8% and 48.9% of the highest activity at low temperatures of 20 and 4 °C, respectively. AlyPM, from *Pseudoalteromonas* sp. SM0524 [24], maintained 19% of the highest activity at 5 °C; TsAly6A, from *Thalassomonas* sp. LD5, showed 21.1% of maximum activity at 10 °C [5]; and AlyS02, from *Flavobacterium* sp. S02, possessed less than 60% of maximum activity at 15 °C [27]. The ability of cold-adaptive enzymes to maintain higher catalytic activity at lower temperatures is related to their loose protein structure, allowing the enzyme to make sufficient conformational changes for a catalysis reaction with lower activation energy. Aly23 presented higher cold adaptability than the other reported alginate lyases, and this property may be related to its flexible protein structure. Cold-adapted alginate lyase is not only an effective tool for the enzymatic hydrolysis of brown algae, but can reduce energy consumption.

To investigate the effect of reaction pH on the activity of Aly23, we incubated Aly23 under different buffers. The optimal pH for Aly23 was 6.0 (Figure 4C) with an acetic acid–sodium acetate buffer. Aly23 exhibited more than 50% of the highest activity in a weakly acidic and neutral pH (5–7.5). Aly23 activity was almost negligible at a pH below 4.5 and above 9.5, suggesting its sensitivity to strong acid and alkali environments. The optimal pH range was similar to that of most reported alginate lyases, with the highest activity at a pH of 6.0–8.0 [21,28,29]. The pH stability of Aly23 was investigated after incubation of the enzyme in a buffer at different pH levels (4.0–10.0) at 4 °C for 24 h. As shown in Figure 4D, Aly23 exhibited the optimal pH stability at pH 5.0–6.0 and retained more than 80% of the initial activity after incubation.

The Michaelis–Menten kinetic constant (K_m_) and k_cat_ for Aly23 were determined using nonlinear fitting (Supplementary Materials, Figure S1). The K_m_ of Aly23 was 3.08 mg/mL. In comparison with other alginate lyases, such as Alg17C (35.2 mg/mL) [30], OacA (3.25 mg/mL) [31], and Alg2A (3.33 mg/mL) [35], Aly23 had a lower K_m_, indicating that Aly23 had a better substrate affinity for alginate. The catalytic efficiency (kcat/Km) of Aly23 was 0.90 mg^−1^ mL s^−1^, which was lower than other PL17 family alginate lyases such as Microbulbifer sp. ALW1 alginate lyase (67.36 mg^−1^ mL s^−1^) [28], OalS6 from *Shewanella* sp. Kz7 (61.7 mg^−1^ mL s^−1^) [3]. 

### 2.4. Effects of Ions on the Activity of Aly23

**Figure 5:**
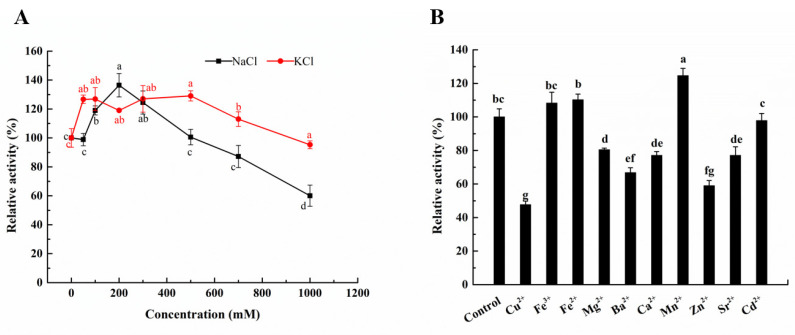
(**A**) Effects of NaCl and KCl on the enzymatic activity of Aly23; (**B**) effects of metal ions on the activity of Aly23. Aly23 activity was determined using various metal ions (namely, NaCl, KCl, CaCl_2_, MgCl_2_, FeCl_2_, CuCl_2_, CoCl_2_, MnCl_2_, BaCl_2_, CdCl_2_, and ZnCl_2_) at the final concentration of 1 mM. Different letters on the bar denote significant differences (*p* < 0.05). Data were represented as the mean ± standard deviation of triplicate measurements.

Alginate lyase derived from marine source often displayed salt adaptability, including salt tolerance and salt activation. AlyS02 reached its maximum value under 500 mM NaCl conditions, and this value was approximately 250% of the relative activity when NaCl was not added [36]. The enzyme activity of AlyPM from *Pseudoalteromonas* sp. SM0524 in 0.5–1.2 M NaCl was six times that of 0 M NaCl [24]. The optimal NaCl concentration for AlgA from *Bacillus* sp. Alg07 activity was 200 mM, but no enzyme activity was detected at 0 mM [37]. The effect of NaCl and KCI on the enzyme activity of Aly23 was investigated. As shown in Figure 5A, Aly23 retained 60% and 95% of the enzyme activity with the addition of 1000 mM NaCl and KCl, respectively, exhibiting great salt tolerance. The enzyme activity of Aly23 was enhanced by 136% with 200 mM NaCl and enhanced by 126% with 300 mM KCl. In comparison with other reported salt-dependent alginate lyases, the enzyme activity of Aly23 only slightly depended on salt addition. Alginate lyases with different salt adaptabilities were adapted to different application conditions. Under low or no salt content, alginate lyases that highly rely on salt often showed very low activity, whereas alginate lyase that did not depend on salt could hydrolyze alginate with high efficiency. It also helped in reducing salt consumption and facilitated subsequent product purification.

The effects of metal ions on the enzyme activity of Aly23 were also studied. As shown in Figure 5B, the enzyme activity of Aly23 could be activated by Fe^3+^, Fe^2+^, and Mn^2+^ and inhibited by Cu^2+^, Mg^2+^, Ba^2+^, Ca^2+^, Zn^2+^ and Sr^2+^. The heavy metal ions Cu^2+^ and Sr^2+^ had a strong inhibitory effect on enzyme activity, possibly because of enzyme denaturation. Ca^2+^ could affect enzymatic activity by noncompetitive inhibition, resulting in the formation of a stable intermediate complex of the enzyme [33]. In addition, in the presence of 1 mM Mn^2+^, the enzyme activity increased by 24.6%. A similar situation was reported in AlgSH17 [21] with an increase of 85.24%. Some alginate lyases could be activated by divalent metal ions. The PL-17 family oligoalginate lyase OalV17, from *Vibrio* sp. SY01 [32], is a divalent metal ion-resistant enzyme. In comparison with OalV17, similar to another characterized family 17 oligoalginate lyase Oal17A [29], Aly23 is also sensitive to metal binding conditions. It is noteworthy that some metal ions may affect the enzymatic activity of alginate lyase through the interaction with the substrate, including promoting the gelatinization of alginates, which decreases the binding efficiency of the enzyme and substrate. When examining the effect of metal ions on the enzymatic activity of alginate lyase, it is vital to consider the chemical interaction of salts and substrates in the reaction mixture.

### 2.5. Structural Insight into the Cold-Adaptation Property of Aly23

The cold-adaptation property of enzymes ensured considerable catalytic efficiency under low temperatures, and it was remarkably influenced by their structural flexibility [7]. At present, although some studies have focused on the cold-adapted alginate lyase from various polysaccharide lyase families [5,24,27,38], the structural mechanism of the cold-adaptation property of alginate lyase has not been studied. In the present study, a comparative molecular dynamic simulation study was implemented to investigate the effect of temperature on the structural flexibility of Aly23. We carried out 50 ns MD simulations of Aly23 at 10 °C and 30 °C and the results were calculated. As shown in Table 2, the structural parameters of the MD simulation of Aly23 under different temperatures—including the root mean squared error (RMSD), root mean square fluctuation (RMSF), radius of gyrate, solvent accessible surface area (SASA), salt bridge, and hydrogen bonds—only had slight variances. Detail plots for these values during the whole simulation process are listed in the Supplementary Materials (Figure S2). The structural flexibility of mesophilic or thermophilic enzymes would largely decrease at lower temperatures and hinder their catalytic ability. Aly23 could retain 60.2% of its highest activity at 10 °C, and exhibited substantial heat-sensitive characteristics, which may be explained by its distinguished maintenance of considerable structural flexibility at low temperatures. It also tended to have major structural disorder at high temperatures.

**Figure 6:**
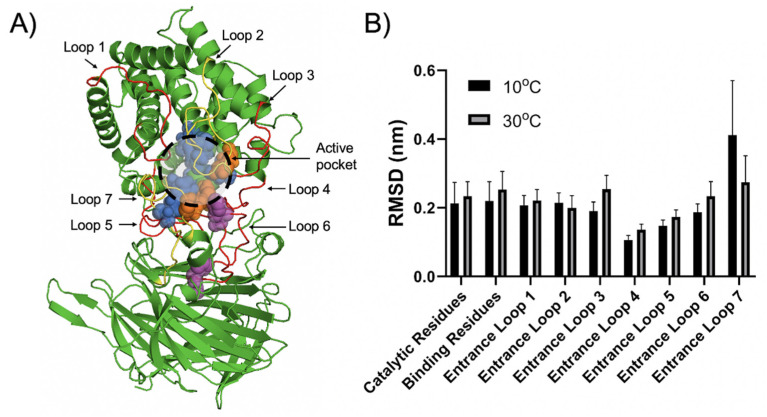
(**A**) Active pocket residues and nearby “entrance” loops of Aly23. Catalytic residues are depicted as orange spheres. Carbohydrate binding residues are depicted as blue spheres. Metal ion-binding residues are displayed as magenta spheres. The “entrance” loops with low RMSD at 10 °C are shown as red lines. The “entrance” loops with high RMSD at 10 °C are shown as yellow lines. (**B**) Average RMSDs of the active pocket residues and nearby “entrance” loops at 10 and 30 °C. The results were obtained by analyzing 50 ns MD simulation trajectories. The average RMSDs and respective deviations were calculated by analyzing the RMSD change during 50 ns MD simulation trajectories. Deviations reflected the size of the change during MD simulation.

To further study the structural basis for the cold adaptability of alginate lyase, we analyzed the conformational dynamic of the active pocket and nearby “entrance” loops of Aly23. The active pocket of Aly23 consisted of H207, Y263 and Y454 as catalytic residues, and Q151, N154, N206, Y262, R265, and R442 as carbohydrate binding residues (Figure 6A). Although the RMSDs of the overall structure at 10 and 30 °C only had slight differences, the average RMSDs of the catalytic and binding residues at 10 °C were 0.021 and 0.034 nm lower than that at 30 °C, respectively, which may explain the decreased activity at 10 °C (Figure 6B and Supplementary Materials, Figure S3). Several “entrance” loops were located near the active pocket of Aly23, including A72-Y86, T131-S152, G196-N206, M249-P261, L305-I325, F408-W425 and V435-P456. These “entrance” loops may affect substrate binding and product release, thereby regulating the catalytic activities of enzymes. Loop engineering of epoxide hydrolase could expand the product releasing tunnel and remarkably enhance the activity [39]. Salt activation of an alginate lyase, Aly1281, could be explained by the stabilization effect of salt ions on the lid loops near the active tunnel [40]. Among these “entrance” loops, most loops were stabilized at low temperatures, whereas some loops exhibited a high RMSD at 10 °C. The average RMSDs of the entrance loop 2 (T131-S152) and entrance loop 7 (V435-P456) at 10 °C were 0.0146 and 0.138 nm higher than that at 30 °C, respectively. The flexibility increases of specific “entrance” loops of Aly23 may compensate for the rigidification of the catalytic-related domains at low temperature, which can be beneficial for the catalytic reaction at low temperatures. In summary, the comparative MD simulation of Aly23 revealed putative conformational dynamic features, including the overall structural flexibility and the possible effect of specific “entrance” loops, which may help in understanding the cold-adaptation property of alginate lyase from the PL-17 family.

### 2.6. Substrate Specificity and Degradation Products of Aly23

**Figure 7:**
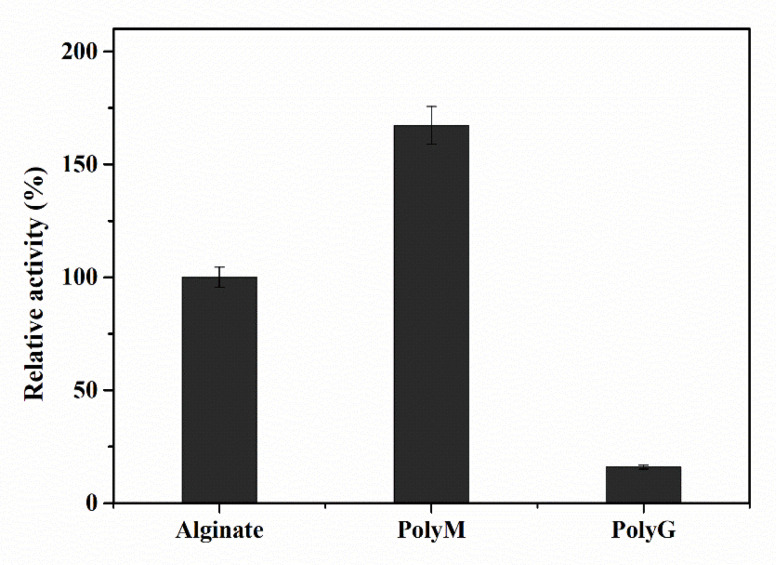
Substrate specificity of the recombinant alginate lyase Aly23. The substrates include sodium alginate, Poly M, and Poly G.

Most of the reported alginate lyases from the PL-17 family exhibit a preference for polyM and display minor activity toward polyG, including: OalC from *Vibrio splendidus* 12B01 [31]; AlgSH17 from *Microbulbifer* sp. SH-1 [14,21]; HyAly-I from *Hydrogenophaga* sp. UMI-18 [25]; OalV17 from *Vibrio* sp. SY01 [32]; AlgL17 from *Microbulbifer* sp. ALW1 [28]; Alg17B from BP-2 [33]; and OalS17 from *Shewanella* sp. Kz7 [3]. However, only a few alginate lyases exhibit similar catalytic activity toward polyM and polyG, such as Oal17A from *Vibrio* sp. W13 [29], OalB from *Vibrio splendidus* 12B01 [31], and OalY1 and OalY2 from *Halomonas* sp. QY114 [34]. To investigate the substrate specificity of the enzyme, we used different substrates for the hydrolysis reaction of Aly23, and the relative activity was calculated. Figure 7 shows the relative activities of Aly23 alginate lyase toward alginate, polyM, and polyG. Aly23 lyase exhibited the highest degradation activity toward polyM, which was 169% of that toward sodium alginate. Only 15.9% of the relative activity was retained for the hydrolysis reaction toward polyG. The results confirmed that Aly23 showed a preference for polyM as a substrate, like most alginate lyase of PL-17 family.

**Figure 8:**
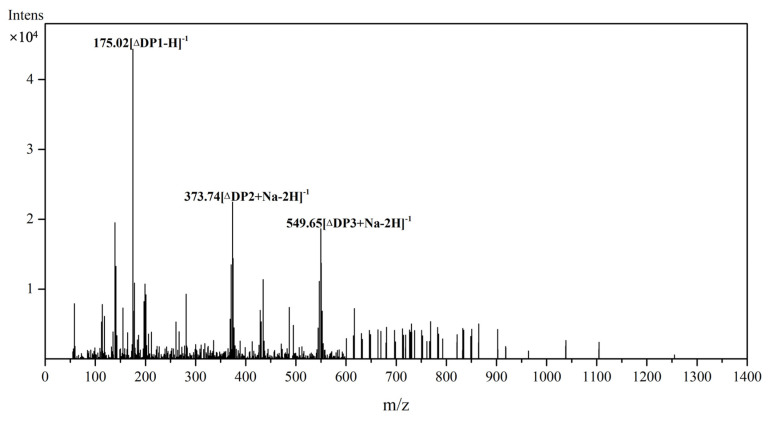
ESI-MS analysis of the enzymatic hydrolyzed products of Aly23 with alginate as substrate.

To determine the degradation products of Aly23 towards sodium alginate, we used electrospray ionization–mass spectrometry (ESI-MS) for the degradation product analysis. The degradation reaction was allowed to proceed for 24 h before the products were collected, purified, and subjected to ESI-MS analysis. As shown in Figure 8, compared with Alg17C [30], Oal17A [29], and OalS17 [3] from the PL-17 family, which only contained monosaccharides, the main products of the Aly23 degradation reaction contained monosaccharides, disaccharides, and trisaccharides. The existence of monosaccharides and disaccharides could infer that Aly23 possessed both exolytic and endolytic activities, which is similar to the two other PL-17 family enzymes, Alg17B [33] and AlgSH17 [21]. Regarding the benefit of monosaccharides and other oligosaccharides in the hydrolysates, it could have great potential in the microbial production of biofuel and in powerful natural antioxidant production.

### 2.7. Antioxidant Function of the Degradation Products of Aly23

**Figure 9:**
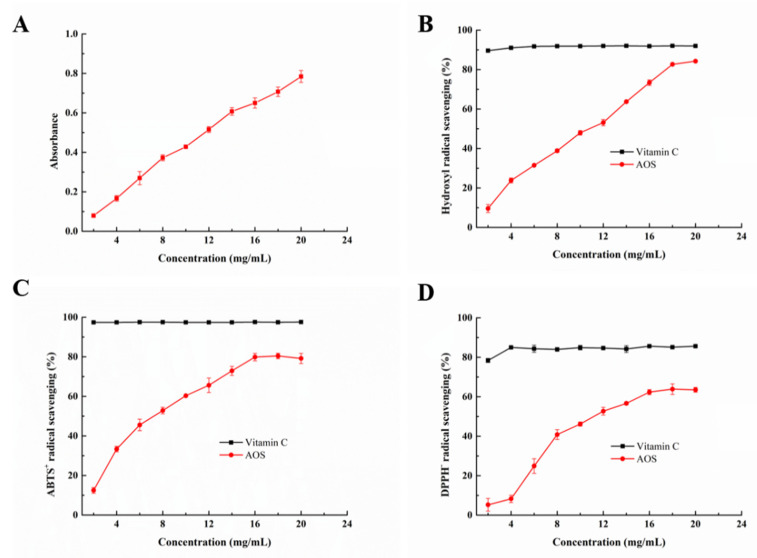
Antioxidant function of alginate oligosaccharides: (**A**) reducing ability; (**B**) scavenging effect on hydroxyl radicals; (**C**) scavenging effect on ABTS radicals; (**D**) scavenging effect on DPPH radicals. Data were represented as the mean ± standard deviation of triplicate measurements.

Considering the excellent antioxidant activities of AOS, it has attracted extensive attention in the food industry [10,41]. In the present study, the antioxidant ability of AOS obtained using Aly23 was investigated using various methods. Antioxidant activities were highly correlated with reducing power [42,43], and this property was normally characterized using Fe^3+^ reduction ability [44]. As shown in Figure 9A, the reducing effects of AOS increased from 0.08 to 0.79 at 700 nm with increasing AOS concentration from 2 mg/mL to 20 mg/mL, indicating a concentration-dependent ability to reduce Fe^3+^. Hydroxyl radicals were mainly formed from high-energy irradiation or a H_2_O_2_ mediated metal-catalyzed process. Its high reactivity may damage biological molecules, resulting in lipid peroxidation, protein oxidation and degradation, or DNA impairment [45]. In the present study, the hydroxyl radica-scavenging activity of the saccharides was evaluated using salicylic acid as molecular probe. Figure 9B shows a concentration-dependent ability to scavenge hydroxyl radicals with a maximum activity of 84% when applied at a concentration of 20.0 mg/mL, and the EC_50_ of the hydrolysates was 10.79 mg/mL. Hydroxyl radicals could probably abstract AOS a carbon-bonded H atom to form a carbon-centered radical and H_2_O [46]. Furthermore, the reducing ability of AOS was generally associated with the presence of reductones, which could exert antioxidant action by breaking free radical chains after donating a hydrogen atom. 2,2′-Azinobis-(3-ethylbenzthiazoline-6-sulphonate) (ABTS) and 2,2-Diphenyl-1-picrylhydrazyl (DPPH) assay are suitable approaches to quantify the antioxidant activity of hydrogen-donating compounds [42]. As shown in Figure 9C,D, AOS was obtained by Aly23-scavenged ABTS^+^ and DPPH free radicals in a concentration-dependent manner, at concentrations lower than 18 mg/mL. AOS (18 mg/mL) showed the highest ABTS^+^ and DPPH radical scavenging activities of 81.95% and 63.83%, respectively. The EC_50_ values of AOS produced by Aly23 (DP = 1–3) for ABTS^+^ and DPPH radicals were 8.90 and 14.07 mg/mL, respectively, which were higher than those of AOS produced by AlgSH17 (DP = 1–6, EC50 for ABTS^+^ = 8.89 mg/mL, EC_50_ for DPPH = 10.70 mg/mL) [14] and Aly1281 (DP = 2, EC_50_ for ABTS^+^ = 5.65 mg/mL and EC_50_ for DPPH = 7.84 mg/mL, respectively) [40]. The high degree hydrolysis of algae could increase the antioxidant activities [47]. The high antioxidant capacity was mainly due to the double bond contained in the AOS, which could cause the addition reaction of free radicals and form stable oligosaccharides and free-radical complex structures [10]. However, the monosaccharides produced by the exolytic hydrolysis of alginate lyase transformed into 4-deoxy-L-erythro-5-hexoseulose uronic acid, which reduced the conjugated alkene acid (the key factor for antioxidant activities of alginate oligosaccharides), and resulted in a decrease in antioxidant activity. Therefore, the AOS produced by Aly1281 exhibited higher antioxidant activity than that of AlgSH17 and Aly23. The PL-17 family alginate lyase with only exolytic activity (e.g., Oal17A [29], OalY1, OalY2 [34], Alg17C [30] and OalS17 [3]) could only produce monosaccharides, which are not suitable for producing an AOS antioxidant. On the other hand, the PL-17 family alginate lyase with both exolytic and endolytic activity had higher application potential for AOS antioxidant production.

## 3. Materials and Methods

### 3.1. Materials

*Pseudoalteromonas carrageenovora* ASY5 was preserved in the College of Food and Biological Engineering of Jimei University. *E. coli* DH5α and BL21 (DE3) were obtained from TAKARA (Beijing, China). The pET-28a(+) vector was obtained from Thermo Fisher Scientific (Shanghai, China). Taq DNA polymerase and polyacrylamide were purchased from TransGen Biotech (Beijing, China). Sodium alginate, polyM, and polyG were obtained from Yuanye BioTechnology (Shanghai, China).

### 3.2. Construction of Recombinant E. coli and Sequence Analysis of Aly23

The Aly23 gene was cloned using the genome of strain ASY5 as a template, with the following primers: 5′-CGCGGATCCATGATGAATTTATCTCGAAG-3′. The reverse primer was 5′-CCGGAATTCCTCCTGAGTATTCTTCAACG-3′. After amplification by PCR, the PCR products were purified and ligated into a pET-28a(+) plasmid with T4 DNA ligase, then transformed into E.coli BL21 (DE3)-competent cells for heterologous expression. Multiple sequence alignment was performed using ESPript (http://espript.ibcp.fr/ESPript/cgi-bin/ESPript.cgi, accessed on 7 December 2021). The secondary structure was based on the SWISS-MODEL. The phylogenetic tree construction was carried out using the Molecular Evolutionary Genetics Analysis (MEGA) program version 7.0, based on the neighbor-joining method.

### 3.3. Expression and Purification of Aly23

The recombinant *E. coli* was inoculated in LB medium containing 50 mg/L Kanamycin at 37 °C and 180 rpm. When the OD_600_ reached 0.6–0.8, 0.05 mmol/L IPTG was added to induce the protein expression at 16 °C and 180 rpm for 20 h. The bacterial cells were collected by centrifugation, and then the harvested cells were sonicated. The supernatants containing crude Aly23 were loaded on a Ni-NTA agarose column after centrifugation at 8000 rpm for 20 min at 4 °C. The washing buffer (300 mM NaCl, 15 mM imidazole, 50 mM NaH_2_PO_4_) was used to eliminate non-specific binding impurities. The recombinant protein was eluted with an elution buffer (300 mM NaCl, 250 mM imidazole, 50 mM NaH_2_PO_4_). The eluted fractions with Aly activity were collected in tubes and analyzed by 12% SDS-PAGE. The eluted fractions with purified Aly were then combined and desalted using a Macrosep Advance Centrifugal Device (cut-off 10 kDa, Pall, East Hills, NY, USA).

### 3.4. Enzymatic Activity Assay

Aly23 (0.2 mL) was incubated with 0.8 mL of sodium alginate (0.5%, pH 8.0), and the reaction was carried out at 35 °C for 40 min. Then, heating in boiling water stopped the reaction. Approximately 1 mL of 3,5-dinitrosalicylic (DNS) solution was added and the mixture was boiled for 10 min. The absorbance of the product was determined at 540 nm. One unit of Aly23 activity was defined as the amount of enzyme required to catalyze the production of 1 μmol of reducing sugar per minute.

### 3.5. Determination of Enzymatic Kinetics of Recombinant Alginate Lyases

The kinetic parameters of Aly23 were determined using different sodium alginate concentrations (0.5–4.5 mg/mL) under standard assay conditions. The Michaelis–Menten K_m_ and k_cat_ of recombinant Aly23 were calculated using non-linear regression analysis on GraphPad Prism 8.

### 3.6. Biochemical Characterization of Aly23

To determine the effect of temperature on Aly23 activity, we determined the activity of alginate lyase from 4 °C to 70 °C. The enzyme activity at optimum temperature was defined as 100% to calculate the relative activity. For the temperature stability test, Aly23 was incubated from 30 °C to 65 °C for 0.5 h and 2 h. Residual enzyme activities were measured under the assay conditions described above and calculated using the initial activity as 100%.

To investigate the effect of pH on Aly23 activity, we measured the enzymes in different buffers (50 mM, pH 4.0–10.0). Relative enzyme activities were calculated using the activity obtained at the optimum pH as 100%. For the pH stability test, Aly23 was incubated in the buffers at 4 °C for 24 h. Residual enzyme activities were measured under the assay conditions described above and calculated using the initial activity as 100%.

The effects of metal ions on Aly23 activity were determined using various metal ions (NaCl, KCl, CaCl_2_, MgCl_2_, FeCl_2_, CuCl_2_, CoCl_2_, MnCl_2_, BaCl_2_, CdCl_2_, and ZnCl_2_) at the final concentration of 1 mM. The effects of salts on Aly23 activity were measured at different concentrations of NaCl or KCl (0–1000 mM). Aly23 was incubated with each metal ion or salt at 4 °C for 1 h. The enzyme activity without metal ions and salts was measured as 100% to calculate the relative activity.

### 3.7. Comparative Study by MD Simulation

The 3D structure of Aly23 was determined using the I-TASSER server (http://zhanglab.ccmb.med.umich.edu/I-TASSER, accessed on 10 November 2021). The threading templates included 4ojz, 7bjt, 4nei and 4ok4. The sequence identities between these templates and Aly23 were 50%, 42%, 51% and 50%. The C- score of the obtained 3D structure of Aly23 was 1.30, which was good for I-TASSER modeling. MD simulations were performed using the Gromacs 2019 package with the AMBER 99SB force field. Solvent molecule modeling was performed using the TIP3P water model. The charges were neutralized using Na^+^ and Cl^−^ in the simulated system. Minimizations were performed with the steepest descent integrator, and the conjugate gradient algorithm successively reached a maximum force below 1000 kJ mol^−1^ nm^−1^ on each atom. Van der Waals interactions were truncated at 10 Å, while electrostatic interactions were truncated at 9 Å by using a twin-range cutoff scheme. The particle mesh Ewald method was applied for handling long-range electrostatic interactions. The bonds that link hydrogen atoms were constrained using a linear constraint solver. The pressure was set at 1.0 atm by using a Parrinello–Rahman barostat with a coupling constant of 2 ps. Simulations were carried out with 2 fs timestep. All MD simulations were equilibrated under a constant-volume (NVT) ensemble for 100 ps and a constant-pressure (NPT) ensemble for 100 ps, and the procedure lasted 50 ns. Simulation trajectories were generated, and physical parameters, such as the RMSD, RMSF, radius of gyration, SASA, salt bridge, and hydrogen bonds were calculated using the standard tools of the GROMACS package or VMD 1.9.3. Protein structure images were obtained using PyMOL.

### 3.8. Substrates Specificity of Aly23

The substrate specificities of purified Aly23 were investigated under the assay conditions described previously. The substrates used include 2 mg/mL sodium alginate, polyM, and polyG. The activities of the recombinant alginate lyases with sodium alginate were defined as 100%.

### 3.9. ESI-MS Analysis of the Degradation Products of Aly23

ESI-MS was used to determine the composition and degree of polymerization (DP) of the degradation products. The substrate solution (5 mg/mL sodium alginate) was hydrolyzed with Aly23 at 35 °C for 24 h. When the reducing sugar concentration became stable, the mixture was boiled for 10 min and centrifuged at 12,000 rpm for 10 min. The supernatant was added with absolute ethanol and incubated at 4 °C for 2 h, followed by vacuum freeze-drying. The final degradation products were injected in the negative-ion mode, as previously reported [40]. 

### 3.10. Electrospray Ionization–Mass Spectrometry (ESI-MS) Analysis of the Degradation Products of Aly23

#### 3.10.1. Hydroxyl Radical Scavenging

Hydroxyl radical scavenging activity was determined as previously described [40]. In the present experiment, 2.0 mL of the oligosaccharide solution to be measured was added with 0.1 mL of FeSO_4_ (9.0 mM), 0.6 mL of deionized water, 0.1 mL of salicylic acid (9.0 mM, dissolved in ethanol), and 0.1 mL of H_2_O_2_ (8.8 mM). After incubation at 37 °C for 30 min, the absorbance at 510 nm was determined using distilled water as a blank and Vc as a positive control. Hydroxyl scavenging activity (%) was calculated using Equation (1) as follows:

Hydroxyl radical scavenging activity (%) = (A_0_ − A _sample_)/A_0_ × l00 (1)

where A_0_ and A _sample_ are the blank absorbance and final absorbances at 510 nm for each sample, respectively.

#### 3.10.2. DPPH Radical Scavenging

DPPH radical scavenging activity was determined using a previous method with minor modifications [9]. In the assay used in the present study, 500 μL of the oligosaccharide solution was mixed with DPPH (500 μL) and incubated for 30 min at room temperature in the dark, with distilled water as a blank and Vc as a positive control. The absorbance was measured at 517 nm. The scavenging activity (%) of DPPH was calculated using Equation (2) as follows:

DPPH radical scavenging activity (%) = (A_0_ − A _sample_)/A_0_ × l00 (2)

where A_0_ and A _sample_ are the blank and final absorbances at 517 nm for each sample, respectively.

#### 3.10.3. Ferric Reducing Power

Ferric reducing power was determined as previously described with trivial modifications [48]. Approximately 300 μL of the oligosaccharide solution to be measured was added to 350 μL of sodium phosphate buffer (pH 7.0, 0.2 M) and 350 μL (1%, *w*/*v*) of [K_3_Fe(CN)_6_]. After 20 min of incubation at 50 °C, the solution was cooled to room temperature, then 350 μL of 10% (*w*/*v*) trichloroacetic acid and 150 μL of 0.1% (*w*/*v*) FeCl_3_ were added. The absorbance of the mixture was measured at 700 nm.

#### 3.10.4. ABTS Radical Scavenging

ABTS radical-scavenging activity was evaluated [49]. Equal amounts of ABTS (7 mM) and K_2_S_2_O_8_ (2.45 mM) were mixed and incubated for 16 h in darkness, diluted with PBS (pH 7.0) to reduce the absorbance at A_734_ nm to 0.700 ± 0.020. A total of 0.1 mL of enzymatic hydrolysate solution was added to 1 mL of ABTS solution and incubated at 37 °C for 15 min with distilled water as a blank and Vc as a positive control. The absorbance of this mixture was measured at 734 nm. The scavenging activity (%) of ABTS was calculated using Equation (3) as follows:

ABTS radical scavenging activity (%) = (A_0_ − A _sample_)/A_0_ × l00 (3)

where A_0_ and A _sample_ are the blank and final absorbances at 734 nm for each sample, respectively.

## 4. Conclusions

In conclusion, a novel cold-adapted PL-17 family alginate lyase, Aly23, from marine bacterium *P. carrageenovora* ASY5, was cloned and characterized. The optimal temperature and pH of Aly23 were 35 °C and 6, respectively. Aly23 showed an excellent cold-adaptation property which could retain 48.93% of the highest activity at 4 °C, indicating its application in specific biotechnology areas and industries. The comparative MD simulation of Aly23 revealed putative conformational dynamic features, which may help in understanding the cold-adaptation property of alginate lyase from the PL-17 family. Moreover, a substrate specificity study showed that Aly23 had a higher preference for polyM and displayed minor activity toward polyG. The degradation products of Aly23 mainly include monosaccharides, disaccharides, and trisaccharides. The antioxidant evaluation showed that the enzymatic hydrolyzed oligosaccharides exhibited competent antioxidant activity, indicating the potential applications of Aly23 in the food industry.

## Figures and Tables

**Table 1:** Characteristics of PL-17 family alginate lyase from different sources.

Enzyme	Source	Optimal Temperature	Optimal pH	Product DP	Ref.
Aly23	*Pseudoaltermonas* sp. ASY5	35 °C	6	1–3	This study
oalS17	*Shewanella* sp. Kz7	50 °C	6.2	1	[3]
HyAly-I	*Hydrogenopahaga* sp. UMI-18	40 °C	6	1–3	[25]
algL17	*Microbulbifer* sp. ALW1	35 °C	8	1–4	[28]
AlgSH17	*Microbulbifer* sp. SH-1	30 °C	7	1–6	[21]
Oal17A	*Vibrio* sp. W13	30 °C	7	1	[29]
Alg17C	*Saccharophagus degradans* 2-40	40 °C	6	1	[30]
OalB	*Vibrio.splendidus* 12B01	30 °C	7	—	[31]
OalC	*Vibrio.splendidus* 12B01	35 °C	7.5	—	[31]
OalV17	*Vibrio* sp. SY01	40 °C	7.2	1	[32]
Alg17B	strain BP-2	40–45 °C	7.5–8.0	1–6	[33]
OalY1	*Halomonas* sp. QY114	45 °C	7.05	1	[34]
OalY2	*Halomonas* sp. QY114	50 °C	6.6	1	[34]

**Table 2:** The average structural parameters of the MD simulation of Aly23 under different temperatures.

Parameter ^a^	Simulation Temperature	
	10 °C	30 °C
RMSD (nm)	0.314 ± 0.052	0.322 ± 0.52
RMSF (nm)	0.131 ± 0.064	0.127 ± 0.055
Radius of gyrate (nm)	2.882 ± 0.013	2.864 ± 0.020
SASA (Å^2^)	33130 ± 283	32473 ± 443
Salt bridge	35 ± 3	34 ± 3
Hbond p-p ^b^	514 ± 11	506 ± 11
Hbond p-w ^b^	1497 ± 24	1454 ± 29
Hbond s-s ^b^	85 ± 5	87 ± 6

^a^ The parameters were analyzed using VMD based on 50 ns MD simulation trajectories. The average parameter values and respective deviations were calculated by analyzing the parameter change during 50 ns MD simulation trajectories. Deviation reflected the size of the change during MD simulation. ^b^ “p”, “w” and “s” indicate protein, water molecule and side chain.

## Supplementary Materials

The following are available online at https://www.mdpi.com/article/10.3390/md20020126/s1, Figure S1. The nonlinear fitting calculation of the kinetic parameters of Aly23. Figure S2. (A) RMSDs of alpha carbon atoms in 50 ns MD simulations as a function of time relative to the initial structure of Aly23 at different temperatures. (B) RMSFs of the alpha carbon atoms in 50 ns MD simulations of Aly23 at different temperatures. (C) Gyrate radius of Aly23 structure in 50 ns MD simulations as a function of time at different temperatures. (D) SASA of Aly23 structure in 50 ns MD simulations as a function of time at different temperatures. (E) Salt bridge number of Aly23 in 50 ns MD simulations as a function of time at different temperatures. (F) Hydrogen bonds of Aly23 in 50 ns MD simulations as a function of time at different temperatures. Figure S3. RMSDs of alpha carbon atoms of catalytic residues (A), binding residues (B) and seven entrance loops (C-I) of Aly23 in 50 ns MD simulations as a function of time relative to the initial structure at different temperatures.
