# Supplementary material for: Biochemical Characterization and Cold-Adaption Mechanism of a PL-17 Family Alginate Lyase Aly23 from Marine Bacterium Pseudoalteromonas sp. ASY5 and Its Application for Oligosaccharides Production
Source: Mar Drugs. 2022 Feb 6;20(2):126. doi: 10.3390/md20020126 (PMC8876620; doi:10.3390/md20020126)
Supplement: Supplementary file 1 [file marinedrugs-20-00126-s001.zip › marinedrugs-1565615-supplementary.pdf]

Supplementary Materials

Biochemical characterization and cold-adaption mechanism of A PL-17 family alginate lyase Aly23 from marine bacterium *Pseudo-altermonas* sp. ASY5 and its application for oligosaccharides production

Xiang Tang [1,†], Chao Jiao [1,†], Yi Wei [1], Xiao-Yan Zhuang [1,2,3], Qiong Xiao [1,2,3], Jun Chen [1,2,3], Fu-Quan Chen [1,2,3], Qiu-Ming Yang [1,2,3], Hui-Fen Weng [1,2,3], Bai-Shan Fang [4], Yong-Hui Zhang [1,2,3,*], An-Feng Xiao [1,2,3,*]

[1] College of Food and Biological Engineering, Jimei University, Xiamen 361021, China;
[2] Fujian Provincial Engineering Technology Research Center of Marine Functional Food, Xiamen, Fujian Province 361021, China;
[3] Xiamen Key Laboratory of Marine Functional Food, Xiamen 361021, China;
[4] College of Chemistry and Chemical Engineering, Xiamen University, Xiamen 361021, China;
* Correspondence: yhz@jmu.edu.cn (Y.-H.Z.), xxaaffeng@jmu.edu.cn (A.-F.X); Tel.: +86-592-6181487 (Y.-H.Z.); +86-592-6180075 (A.-F.X.)
† These authors contributed equally to this paper.

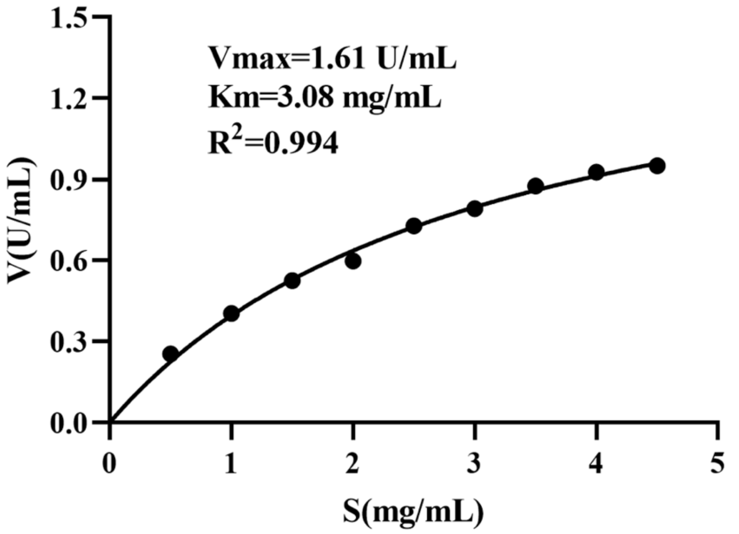

Figure S1. The nonlinear fitting calculation of the kinetic parameters of Aly23.

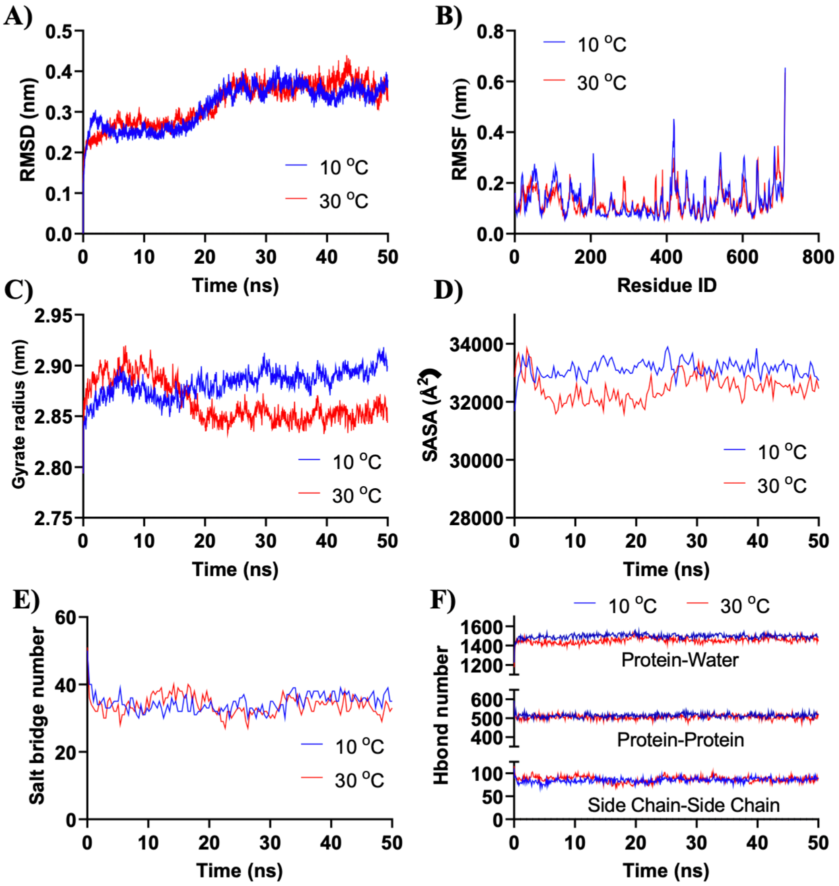

Figure S2. (A) RMSDs of alpha carbon atoms in 50 ns MD simulations as a function of time relative to the initial structure of Aly23 at different temperatures. (B) RMSFs of the alpha carbon atoms in 50 ns MD simulations of Aly23 at different temperatures. (C) Gyrate radius of Aly23 structure in 50 ns MD simulations as a function of time at different temperatures. (D) SASA of Aly23 structure in 50 ns MD simulations as a function of time at different temperatures. (E) Salt bridge number of Aly23 in 50 ns MD simulations as a function of time at different temperatures. (F) Hydrogen bonds of Aly23 in 50 ns MD simulations as a function of time at different temperatures.

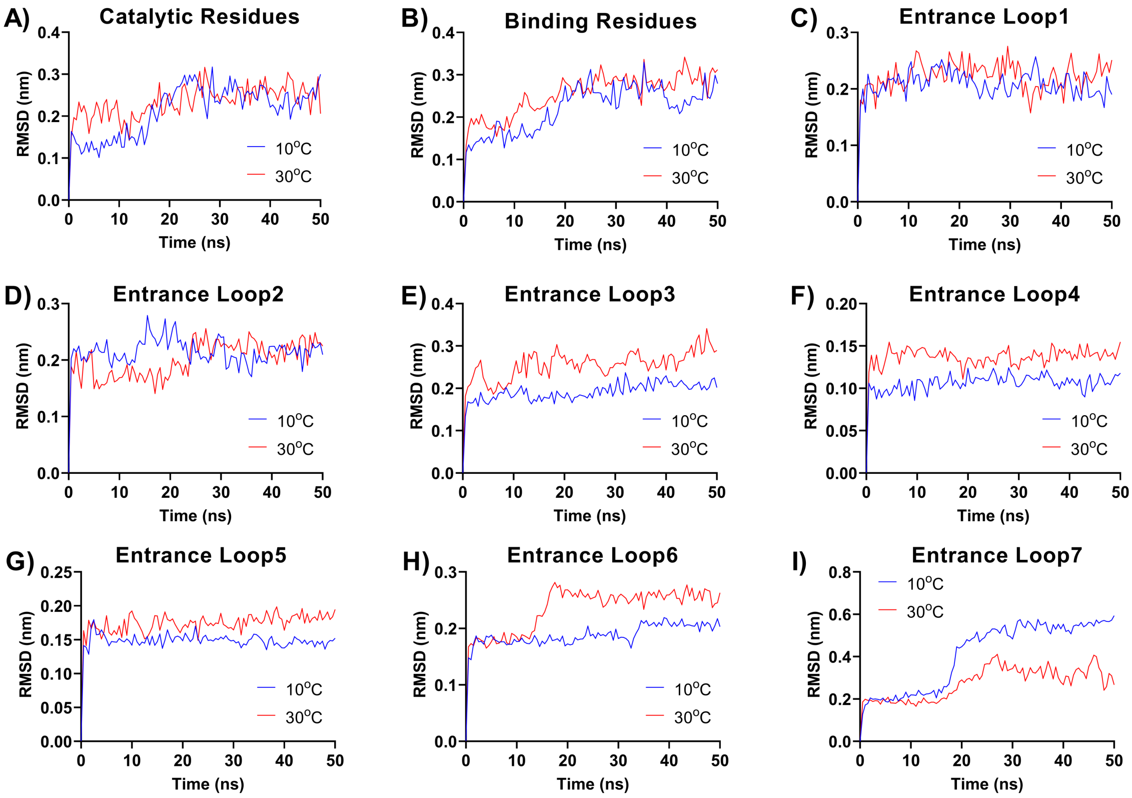

Figure S3. RMSDs of alpha carbon atoms of catalytic residues (A), binding residues (B) and seven entrance loops (C-I) of Aly23 in 50 ns MD simulations as a function of time relative to the initial structure at different temperatures.
